# Cross-Modal Sensory Boosting to Improve High-Frequency Hearing Loss: Device Development and Validation

**DOI:** 10.2196/49969

**Published:** 2024-02-09

**Authors:** Izzy Kohler, Michael V Perrotta, Tiago Ferreira, David M Eagleman

**Affiliations:** 1 Neosensory, Los Altos, CA, United States; 2 Department of Psychiatry, Stanford University, Stanford, CA, United States

**Keywords:** audiology, hearing, high-frequency, wristband, develop, development, wearable, wearables, machine learning, phoneme, phonemes, hear, vibrotactile, vibration, vibrations, sound, sounds, hearing loss, loud noise, loud noises, noise pollution, hearing aids, hearing aid

## Abstract

**Background:**

High-frequency hearing loss is one of the most common problems in the aging population and with those who have a history of exposure to loud noises. This type of hearing loss can be frustrating and disabling, making it difficult to understand speech communication and interact effectively with the world.

**Objective:**

This study aimed to examine the impact of spatially unique haptic vibrations representing high-frequency phonemes on the self-perceived ability to understand conversations in everyday situations.

**Methods:**

To address high-frequency hearing loss, a multi-motor wristband was developed that uses machine learning to listen for specific high-frequency phonemes. The wristband vibrates in spatially unique locations to represent which phoneme was present in real time. A total of 16 participants with high-frequency hearing loss were recruited and asked to wear the wristband for 6 weeks. The degree of disability associated with hearing loss was measured weekly using the Abbreviated Profile of Hearing Aid Benefit (APHAB).

**Results:**

By the end of the 6-week study, the average APHAB benefit score across all participants reached 12.39 points, from a baseline of 40.32 to a final score of 27.93 (SD 13.11; N=16; *P*=.002, 2-tailed dependent *t* test). Those without hearing aids showed a 10.78-point larger improvement in average APHAB benefit score at 6 weeks than those with hearing aids (*t*_14_=2.14; *P*=.10, 2-tailed independent *t* test). The average benefit score across all participants for ease of communication was 15.44 (SD 13.88; N=16; *P*<.001, 2-tailed dependent *t* test). The average benefit score across all participants for background noise was 10.88 (SD 17.54; N=16; *P*=.03, 2-tailed dependent *t* test). The average benefit score across all participants for reverberation was 10.84 (SD 16.95; N=16; *P*=.02, 2-tailed dependent *t* test).

**Conclusions:**

These findings show that vibrotactile sensory substitution delivered by a wristband that produces spatially distinguishable vibrations in correspondence with high-frequency phonemes helps individuals with high-frequency hearing loss improve their perceived understanding of verbal communication. Vibrotactile feedback provides benefits whether or not a person wears hearing aids, albeit in slightly different ways. Finally, individuals with the greatest perceived difficulty understanding speech experienced the greatest amount of perceived benefit from vibrotactile feedback.

## Introduction

Hearing loss affects 466 million people worldwide [1]. High-frequency hearing loss is one of the most common types of hearing loss and renders high-pitched sounds, such as the voices of women and children, more difficult to hear [2,3]. It can affect people of any age but is more common among older adults and people who have been repeatedly exposed to loud noises [4-6]. This type of hearing loss can be frustrating and disabling, making it difficult to understand speech communication and interact effectively with the world, leading to a decline in quality of life and isolation [6,7].

Individuals with high-frequency hearing loss struggle to hear consonants with higher-frequency sound components, such as *s*, *t*, and *f*. As a result of the hearing loss, speech is reported as sounding muffled, most noticeably in noisy environments. Commonly, people with high-frequency hearing loss will report that they can hear but cannot understand [8]. It is often noticed when a person has trouble understanding women’s and children’s voices and detecting other sounds such as the ringing of a cell phone or the chirping of birds. Assistive hearing technologies such as hearing aids and cochlear implants can offer some assistance with understanding speech communication, but they have limitations. One of the most commonly reported disappointments among users of hearing aids and cochlear implants is that they still cannot understand speech, especially in complex environments [9,10].

To address the speech understanding limitations associated with high-frequency hearing loss, we have developed a vibrotactile sensory substitution solution in the form of a wristband [11-13]. This device delivers spatially unique vibrations to the wrist in correspondence with target phonemes that are commonly difficult for individuals with presbycusis to detect. The wristband receives sound from the environment through an onboard microphone and uses a machine learning algorithm to filter background noise (BN) and extract target phonemes from speech. Each phoneme signal is mapped to its own unique linear resonant actuator (LRA) in the strap of the wristband where it is felt as a vibration on the skin. There are four LRAs embedded within the wristband strap, giving each target phoneme a unique spatial location on the wrist. Parts of speech that are audible to the user are unconsciously integrated with the spatially unique vibratory signals representing the inaudible portions of speech. The user is then able to understand a complete and meaningful message through the integration of the complementary sensory inputs [11-13].

Our prior work in this area demonstrated that when two words are algorithmically translated into spatiotemporal patterns of vibration on the skin of the wrist, they are distinguishable to individuals who are hard of hearing or deaf up to 83% of the time for two words that are similar and up to 100% of the time for two words that are not similar [12,14]. Further studies showed that sound-to-touch sensory substitution devices may help people with hearing impairments, allowing them to access sensory information that is otherwise inaccessible. Weisenberger and Russell [15] used single-channel vibrotactile aids designed to translate acoustic stimuli into representative vibration patterns on the wrist to improve performance on environmental sound identification tests from 55% to 95% correct and improve performance on single word identification testing from 60% to 90%.

In this study, we aimed to demonstrate that a simple wearable sensory substitution device that transforms speech sounds into haptic vibrations on the wrist can help individuals with high-frequency hearing loss perceive a greater ability to understand speech communication throughout their normal daily routine. With further development and refinement, this technology has the potential to improve the quality and productivity of their daily interactions, enable them to enjoy audio-based entertainment such as movies and podcasts, help them understand conversations in complicated acoustic environments, and fill the residual gaps of impairment left by their hearing aids.

## Methods

### Participants

Participants were recruited via web-based advertising for a paid study related to hearing loss. Eligibility required (1) an age between 18 and 80 years, (2) having access to a mobile device (iOS or Android) and a computer, (3) English as a primary spoken language, and (4) meeting the following criteria for high-frequency hearing loss: a pure-tone audiogram (either from an audiologist in the past 24 mo or from 2 audiogram mobile apps, Mimi and Hearing & Ear Age Test) must show at least 55 dB of hearing loss at 4 kHz averaged across both ears (with neither ears’ 4-kHz threshold being less than 40 dB of hearing loss) and no more than 35 dB of hearing loss averaged across both ears and across 500-Hz and 1000-Hz tones. These specifications were chosen to capture individuals with hearing loss profiles in alignment with high-frequency hearing loss. Candidates who did not have an audiogram from an audiologist were required to provide audiograms from both audiogram mobile apps, which have been demonstrated as comparable to in-clinic testing [16].

**Figure 1.**
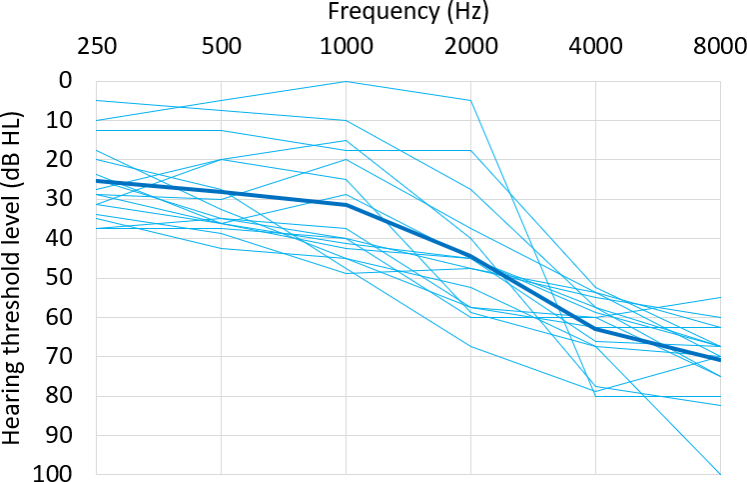
Average pure-tone audiogram of both ears. The thin lines represent each participant; the thick line represents the group average. HL: hearing loss.

A total of 16 eligible participants completed the study: 10 male participants, 5 female participants, and 1 nonbinary participant. The average age was 68.8 (SD 11.6) years. The type and severity of hearing loss were determined from pure-tone audiograms. A total of 9 participants provided audiograms from an audiologist and 7 provided audiograms from the two mobile apps. The average pure-tone threshold of both ears at 500 Hz and 1000 Hz was 30 (SD 13) dB and the average pure-tone threshold of both ears at 4000 Hz was 63 (SD 9) dB of hearing loss (Figure 1). Demographic data for the participants is shown in Table 1.

**Table 1.** Demographic data.

	Age (y)	Sex	Hearing aids	Years with hearing loss	Audiogram source^a^	Hearing loss (dB)^b^											
						250		500		1000		2000		4000		8000	
						R^c^	L^d^	R	L	R	L	R	L	R	L	R	L
B1	64	Male	No	4	Audiologist	10	15	10	15	15	20	15	20	50	55	55	80
B2	75	Male	Yes	15	Audiologist	20	35	15	25	15	15	30	50	80	75	80	85
B3	72	Nonbinary	Yes	22	Audiologist	10	25	25	40	45	45	50	55	65	70	100	100
B4	69	Female	No	35	Audiologist	35	35	45	40	45	45	55	60	55	65	55	55
B5	74	Female	No	15	Mobile app	30	28	33	28	20	20	40	35	55	53	70	70
B6	78	Female	Yes	10	Mobile app	20	20	28	28	48	48	63	73	78	80	70	70
B7	27	Male	Yes	10	Audiologist	10	10	5	5	0	0	5	5	80	80	80	80
B8	73	Male	No	3	Mobile app	33	30	38	35	40	45	45	45	58	60	70	65
B9	68	Male	Yes	15	Mobile app	33	25	40	33	45	38	48	43	58	58	70	65
B10	67	Female	Yes	10	Mobile app	35	33	43	35	50	48	48	48	55	53	55	70
B11	76	Male	Yes	25	Mobile app	33	30	18	23	13	38	58	60	68	68	70	70
B12	66	Female	No	5	Audiologist	25	25	35	35	40	40	60	60	60	60	80	70
B13	79	Male	Yes	15	Audiologist	40	35	35	35	40	35	65	50	65	60	65	60
B14	67	Male	Yes	10	Audiologist	5	5	5	10	10	10	25	30	55	60	75	75
B15	74	Male	No	5	Mobile app	28	20	43	30	33	25	45	45	65	68	65	70
B16	71	Male	No	20	Audiologist	40	35	40	35	40	40	45	50	50	60	60	60

a Audiogram source indicates where the audiogram originated from. Audiologist indicates the audiogram was measured by an audiologist, and mobile app indicates the participant provided two audiograms measured by the Mimi and Hearing & Ear Age Test mobile apps.

b Decibels of hearing loss at 7 pure tones in the left and right ears. Hearing loss values are measured without cochlear implants or hearing aids. Note, 90 dB of hearing loss is the most the test can detect.

c R: right.

d L: left.

### Device

**Figure 2.**
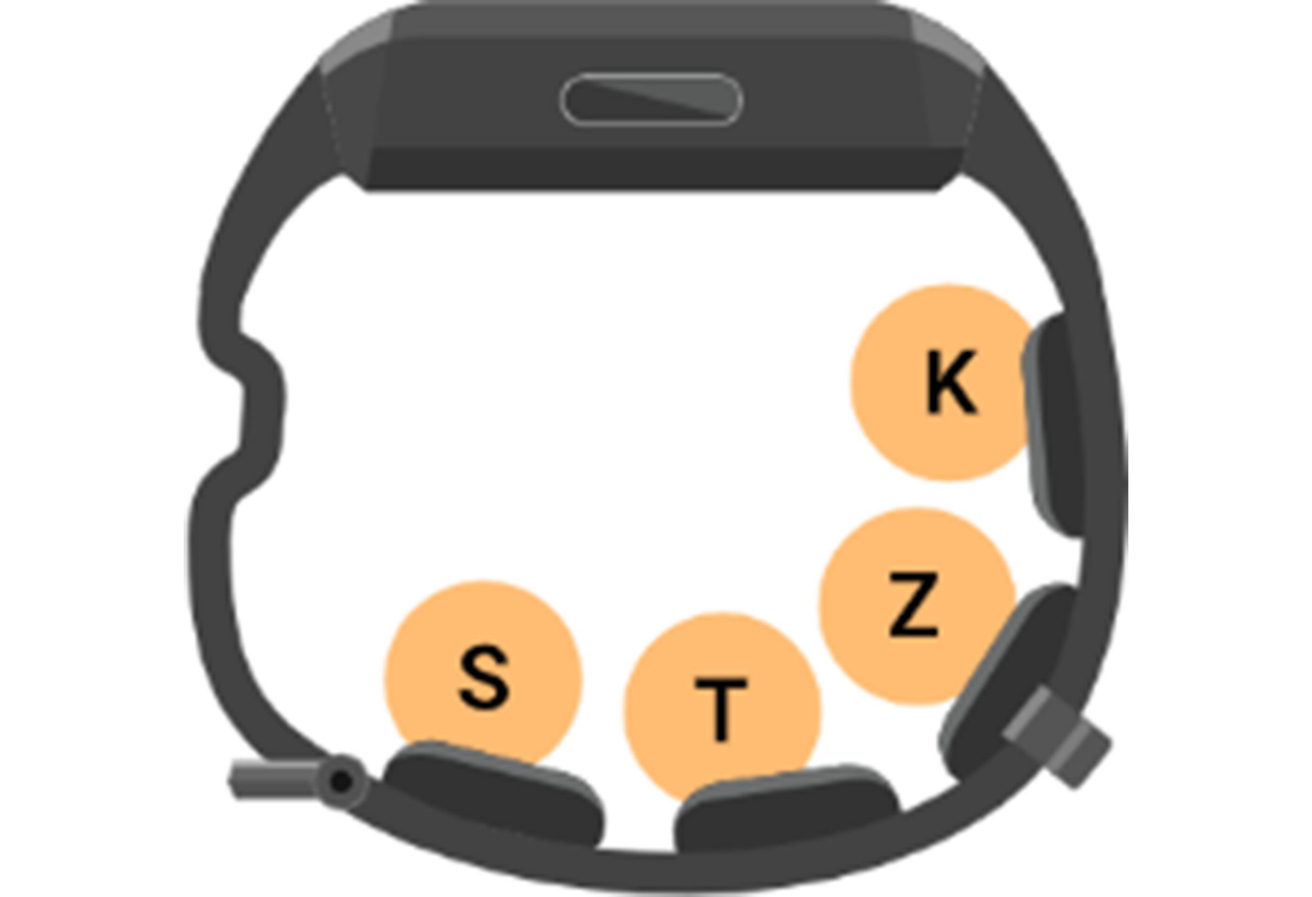
The Neosensory wristband has four vibrating motors embedded in the wrist strap. The top of the wristband contains a power button and a microphone. Each phoneme is assigned to an independent motor.

Participants wore a haptic wristband (Figure 2) that vibrated to indicate the occurrence of specific phonemes. The wristband contained four vibrating motors embedded in the wrist strap, a microphone, a power button, a microcontroller, and a battery.

The motors were LRAs that vibrated in a sine wave and were capable of rising from 0% to 50% of their maximum amplitude within 30 milliseconds. The motors vibrated at 175 Hz, the frequency at which human skin has the highest sensitivity [17]. Each motor vibrated at 1.7 GRMS (root mean squared acceleration from gravity; 16.6 m/s^2^). The motors were separated from one another at a distance of 18.2 mm and 19.2 mm for the small and large wristband sizes, respectively (center-to-center distances). Each motor pad contacted the wearer’s skin on a rectangular area that measured approximately 8.2 mm by 8.5 mm.

The top of the wristband was a module that contained the power button, a microphone, and a microcontroller. The microphone captured audio and sent this data to the microcontroller. The microcontroller processed the audio data through a phoneme-detection algorithm and vibrated the motors according to the output of the algorithm. Additional microphone characteristics are provided in Multimedia Appendix 1.

### Algorithm

The algorithm processed incoming audio to determine when any target phoneme was detected. If a target phoneme was detected, the corresponding motor vibrated for 80 ms.

The four target phonemes were /s/, /t/, /z/, and /k/. Each motor on the wristband was assigned to a different target phoneme. Figure 2 shows the motor assignments for each phoneme. The four phonemes were chosen based on a combination of the following three factors: (1) how difficult each phoneme is for hearing-impaired listeners to hear, (2) how frequently each phoneme occurs in spoken English, and (3) how well our algorithm can detect each phoneme. The difficulty was pooled from several studies of phoneme confusion for hearing-impaired listeners. Phatak et al [18] asked older hearing-impaired listeners to identify the consonant in a presented consonant-vowel syllable. Woods et al [19] presented the California Syllable Test, which uses consonant-vowel-consonant syllables, to older hearing-impaired listeners in both aided (with hearing aids) and unaided conditions. Sher and Owens [20] presented a four-alternative forced-choice test with consonant-vowel-consonant syllables, where either the initial or final consonant differed between choices. Synthesizing the results of these three studies, we found that the following consonants are the most difficult to hear for a listener with presbycusis: /dh/, /th/, /ng/, /v/, /b/, /hh/, /f/, /z/, /s/, and /t/. Of these, /th/ and /ng/ are present in spoken English less than 1% of the time [21]. Our algorithm performed poorly on /dh/, /b/, /f/, and /hh/.

#### Phoneme Detection

The phoneme detection algorithm was trained using the elastic compute cloud on Amazon Web Services. The training data consisted of a combination of pure LibriSpeech and LibriSpeech rerecorded through the onboard microphone on the wristband. LibriSpeech is a corpus of approximately 1000 hours of English speech with standard American accents sampled at 16 kHz that has been shown to produce excellent performance in speech recognition models trained with it [22]. To produce a corpus of English read speech suitable for training speech recognition systems, LibriSpeech aligns and segments audiobook read speech with the corresponding book text automatically and then filters out portions with noisy transcripts. The purpose of using rerecorded data was to tune the algorithm’s parameters to speech sounds representative of those it would encounter from the wristband’s microphone.

The algorithm consisted of feature extraction and inference engine components. The feature extraction module segmented an audio stream captured from the microphone into 32-millisecond frames with 16 milliseconds of overlap. Each audio frame underwent analysis to extract distinct features suitable for phoneme recognition. The features were also subject to further processing that amplified phoneme-specific information contained and ensured robustness toward continuously changing environmental conditions.

The inference engine took these feature vectors and output phoneme predictions. The core of the inference engine was a neural network model that used a real-time temporal convolutional network structure optimized for real-time speech recognition. The full latency from phoneme onset to vibration onset was 170 milliseconds. The algorithm performance is shown below in Table 2.

**Table 2.** Algorithm performance.

	Precision^a^	Recall^b^	*F*_1_-score^c^
K	0.86	0.75	0.8
S	0.86	0.89	0.88
T	0.85	0.65	0.74
Z	0.86	0.72	0.78
Macroaverage	0.86	0.75	0.8

a Precision is the ability of a classification model to return only the data points in a class. It is calculated by dividing the true positives by the sum of the true positives and false positives.

b Recall is the ability of a classification model to identify all data points in a relevant class. It is calculated by dividing the true positives by the sum of the true positives and false negatives.

c *F*_1_-scores are a single metric that combines recall and precision using the harmonic mean. It is calculated by dividing the true positives by the sum of the true positives plus half of the sum of the false positives and false negatives.

### Paradigm

Participants wore the wristband every day for 6 weeks. Each day the participants were required to spend at least 1 hour watching television or listening to an audiobook, podcast, or other speech-based media while wearing the wristband and not wearing earbuds or headphones. The instructions were to choose something engaging so their attention would be directed toward understanding what was being said, while the wristband provided the assistive haptic feedback. No further guidelines were enforced for distance from the audio source or volume. The purpose of this required daily exercise was to ensure the participant was immersed in a minimum amount of active listening each day so the brain would learn to integrate the audible speech sounds with the haptic vibratory representations of the inaudible speech sounds to form a complete meaning. In addition to the required hour of practice, participants were encouraged to wear the wristband whenever engaged in conversation or active listening to speech communication.

### Tasks

#### Abbreviated Profile of Hearing Aid Benefit

Before starting the study and at the end of each week during the study, participants completed a modified version of the Abbreviated Profile of Hearing Aid Benefit (APHAB) that did not include 6 questions related to the aversiveness subscale [11]. These questions were removed because they ask about the unpleasantness of sounds heard through a hearing aid, which does not apply to our device. The remaining 18 questions on the APHAB ask questions about one’s ability to understand verbal communication in different scenarios. For example, one of the questions is “When I am in a crowded grocery store, talking with the cashier, I can follow the conversation.” In the conventional questionnaire, participants answer the questions independently about their experiences while using and while not using their hearing aids. In this study, participants answered the questions independently about their experiences while using and while not using the wristband. If the participant regularly wore hearing aids, “with the wristband” referred to wearing the wristband in addition to their hearing aids, and “without the wristband” referred to wearing their hearing aids alone. The test was administered through a web-based questionnaire that captured the data onto a datasheet for analysis. The benefit score is calculated by subtracting the final aided score at the conclusion of the trial from the baseline unaided score that was measured at the beginning of the trial. Lower raw APHAB scores indicate lower levels of disability associated with hearing loss. Higher benefit scores indicate more perceived benefits from the intervention.

#### Final Questionnaire

On the final day of the study, participants answered a questionnaire that asked 2 questions using a Likert scale from 1 to 10: “How much did the Clarify wristband help you understand speech?” and “How likely are you to recommend the Clarify wristband to a friend or colleague?”

### Ethical Considerations

The study protocol was approved by Solutions IRB (Protocol #2016/01/7), an independent institutional review board accredited by the Association for the Accreditation of Human Research Protection Programs, Inc. All participants gave written informed consent following the Declaration of Helsinki. Upon completion of the study, participants were given a US $100 Amazon gift card for their time. At the conclusion of the study, all data were deidentified to safeguard participant information.

## Results

**Figure 3.**
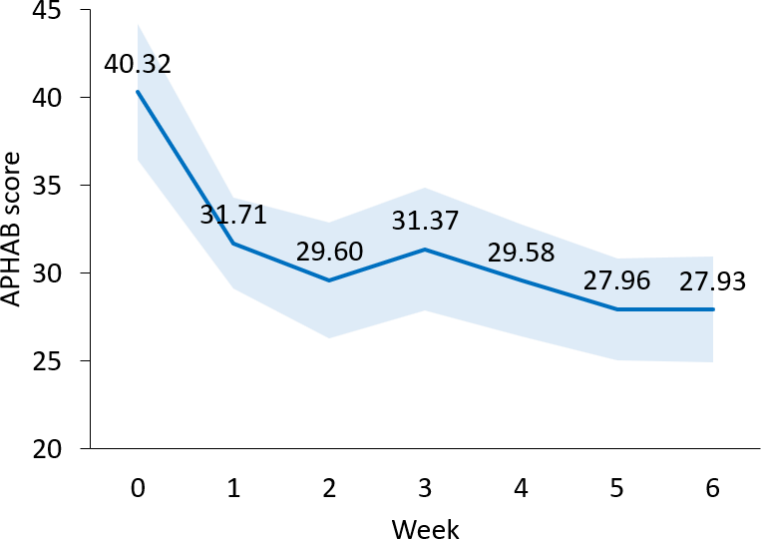
Six-week progression of the APHAB scores. Error boundary (light blue) represents SE of the mean. Week 0 score is the unaided APHAB score (before starting with the wristband); subsequent weeks show the aided APHAB score with the wristband. APHAB: Abbreviated Profile of Hearing Aids Benefit.

**Figure 4.**
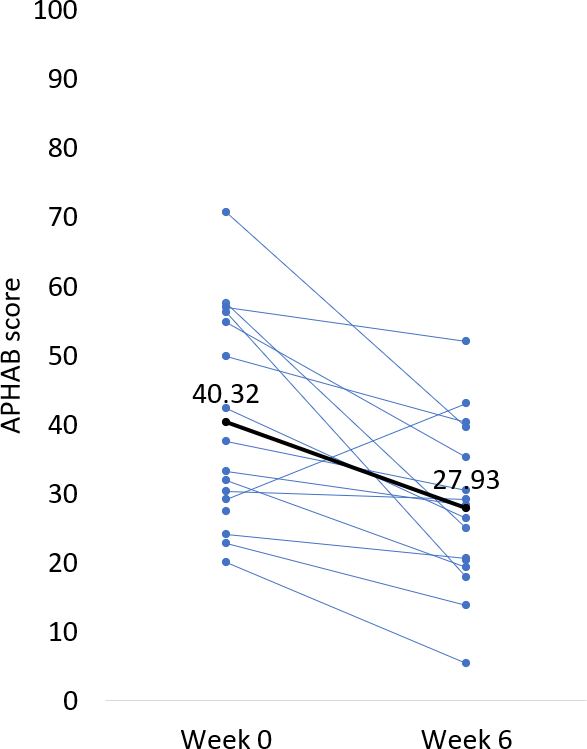
Individual baseline and week 6 APHAB scores. Thin lines represent each participant, and the thick line represents the group average. APHAB: Abbreviated Profile of Hearing Aids Benefit.

As shown in Figure 3 and Multimedia Appendix 2, after only 1 week of wearing the wristband daily, the average APHAB benefit score (*unaided – aided*) was 8.61 points, with a baseline score of 40.32 points that dropped to 31.71 points (SD 12.11; N=16; *P*=.01, 2-tailed dependent *t* test). Baseline was defined as the unaided APHAB score taken before starting to use the wristband. As a reminder, if the participant regularly used hearing aids, they were asked to answer the unaided questions based on how they felt with their hearing aids on. If the participants never used hearing aids, they were asked to answer the unaided questions based on how they felt without any hearing assistance. The average aided APHAB score continued to trend down for the remaining 5 weeks of the study. By the end of the 6-week study, the average APHAB benefit score had reached a clinically meaningful and statistically significant value of 12.39 points [23] from a baseline of 40.32 to a final score of 27.93 (SD 13.11; N=16; *P*=.002, 2-tailed dependent *t* test). Individual data is presented in Figure 4.

**Figure 5.**
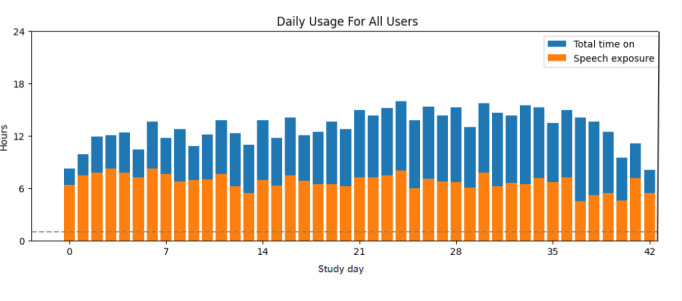
Daily use for all participants. Bar height represents the total time the wristband was on. Orange represents the portion of time the wristband detected the presence of speech sounds. The dotted line represents the 1-hour minimum that participants were instructed to be around speech.

Time wearing the wristband and time exposed to speech were verified through the collection of data from backend logging that records when the wristband is turned on or off and when a phoneme is detected. As seen in Figure 5, participants wore the wristband for an average of 12.9 (SD 8.1) hours per day and were exposed to speech for an average of 6.7 (SD 3.3) hours per day.

**Figure 6.**
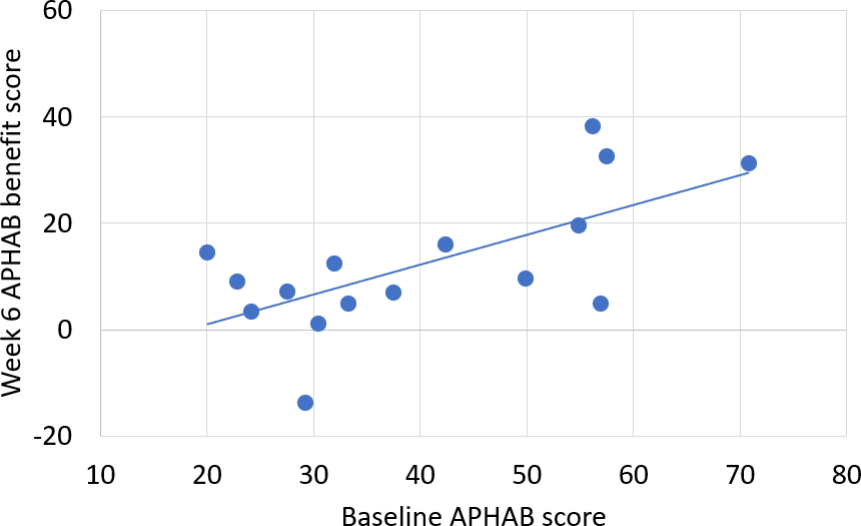
The baseline APHAB score correlates with the final APHAB benefit score. The linear regression demonstrates the correlation between the degree of disability without the assistance of Clarify at baseline and the final benefit score at week 6 with the aid of Clarify. APHAB: Abbreviated Profile of Hearing Aid Benefit.

Simple linear regression analysis was used to test if a participant’s baseline APHAB score explains their benefit APHAB score after 6 weeks, indicating that those with greater subjective difficulty understanding speech may stand to benefit the most from the haptic assistance of the wristband (Figure 6). The results of the regression indicate that the average baseline score explains 43% of the variation in the average APHAB benefit score at 6 weeks (*F*_1,14_=10.55; *P*=.006). These results are significant at the *P*<.05 level.

**Figure 7.**
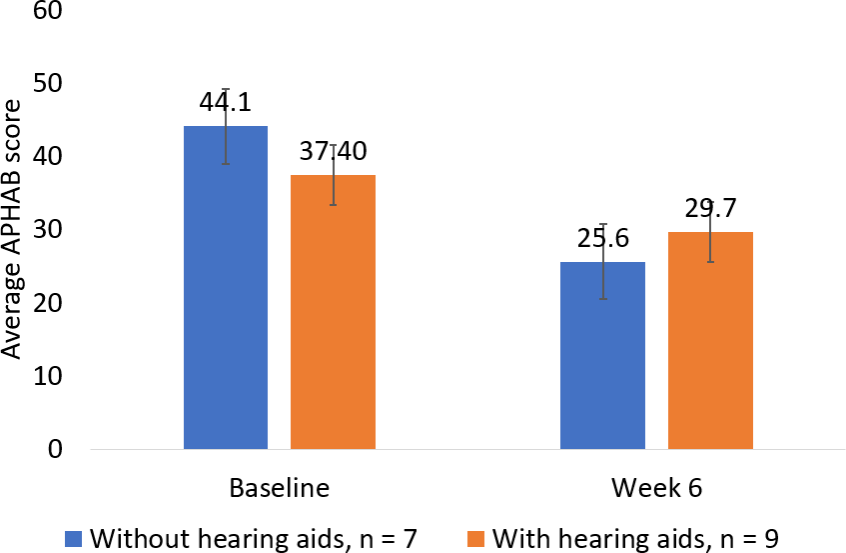
Non–hearing aid users ended the study with a higher benefit score than regular users of hearing aids. Error bars represent SE of the mean (SEM). Baseline SEM without hearing aids: 5.83. Baseline SEM with hearing aids: 4.59. Week 6 SEM without hearing aids: 4.38. Week six SEM with hearing aids: 3.78.

We compared participants who used hearing aids to those who did not. A total of 9 participants used hearing aids to help them understand speech, and 7 of the participants did not. Results showed a 10.78 point greater APHAB benefit score at 6 weeks for participants who did not use hearing aids than for participants who did (*t*_14_=2.14; *P*=.10, 2-tailed independent *t* test; Figure 7). While the difference in the benefit score between the two subgroups was not statistically significant, it did reach the 10-point threshold for clinical relevance [23,24]. The small sample size rendered the study underpowered to detect this difference at *P*<.05, and further study is necessary to validate this finding. Additionally, while the subgroup without hearing aids started the study at a higher level of disability, they ended the study at a lower level of disability than those with hearing aids. The subgroup without hearing aids started with a baseline APHAB score of 44.09 (SD 16.66) points, while the subgroup with hearing aids started with a baseline score of 37.40 (SD 14.61) points. The subgroup without hearing aids concluded the study with an APHAB score of 25.63 (SD 12.51) points, while the subgroup with hearing aids concluded the study with an APHAB score of 29.72 (SD 12.01) points. Another noteworthy difference between the subgroups was that the group who did not wear hearing aids demonstrated both a statistically significant and clinically meaningful aided APHAB benefit score from baseline, while the subgroup that did wear hearing aids did not. The subgroup that did not wear hearing aids ended the study with an average APHAB benefit score from baseline of 18.45 points (SD 11.70 points; n=7; *P*=.005, 2-tailed dependent *t* test). The subgroup that wore hearing aids ended the study with an average APHAB benefit score from baseline of 7.67 points (SD 12.730 points; n=9; *P*=.11, 2-tailed dependent *t* test).

**Figure 8.**
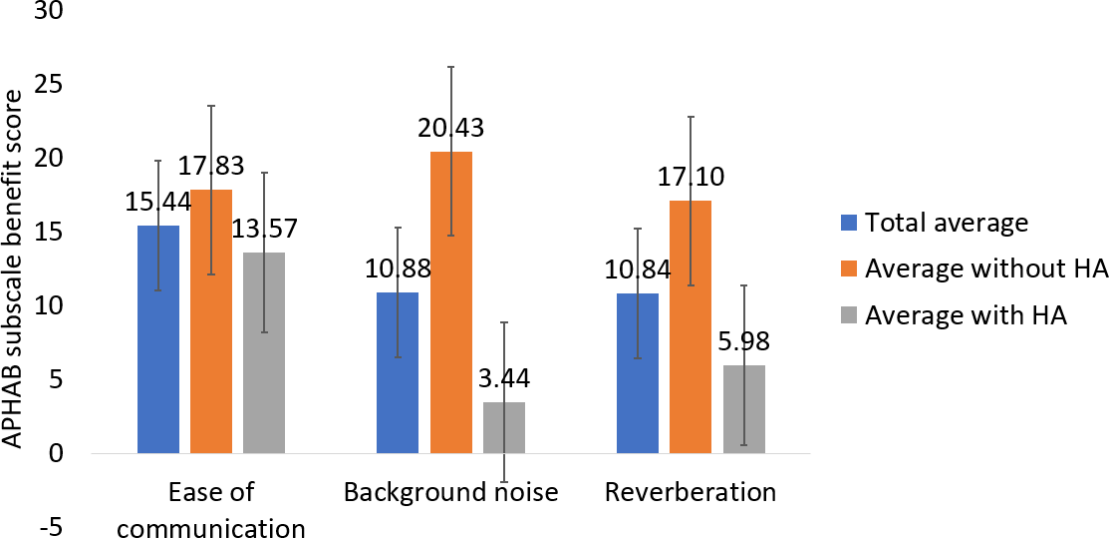
APHAB subscale benefit scores at 6 weeks. Blue bars represent the entire participant group, orange bars are the subgroup who were regular users of hearing aids, and gray bars are the subgroup that did not wear hearing aids. There were 16 participants total, 9 who were regular users of hearing aids, and 7 who did not use hearing aids. Error bars represent the SE of the mean (SEM). Ease of communication SEM for total average: 3.47. Background noise SEM for total average: 4.38. Reverberation SEM for total average: 4.24. Ease of communication SEM for without hearing aids: 4.48. Background noise SEM for without hearing aids: 5.71. Reverberation SEM for without hearing aids: 6.03. Ease of communication SEM for with hearing aids: 5.24. Background noise SEM for with hearing aids: 5.41. Reverberation SEM for with hearing aids: 5.65. APHAB: Abbreviated Profile of Hearing Aid Benefit; HA: hearing aid.

Subscale analyses were performed for ease of communication (EOC), BN, and reverberation (Figure 8 and Multimedia Appendix 3). These subscales are reflective of speech communication under ideal conditions, in noisy environments, and in reverberant environments [23]. The average benefit score for EOC was 15.44 (SD 13.88; N=16; *P*<.001, 2-tailed dependent *t* test). Those who wore hearing aids and those who did not wear hearing aids had similar EOC benefit scores (*t*_14_=2.18; *P*=.60, 2-tailed independent *t* test). The average EOC benefit score for those with hearing aids was 13.57 (SD 15.71; n=9; *P*=.03, 2-tailed dependent *t* test), and the average EOC benefit score for those without hearing aids was 17.83 (SD 11.85; n=7; *P*=.01, 2-tailed dependent *t* test). The average benefit score for BN was 10.88 (SD 17.54; N=16; *P*=.03, 2-tailed dependent *t* test). The average BN benefit score for those without hearing aids was 16.99 points higher than those with hearing aids (*t*_14_=2.14; *P*=.05, 2-tailed independent *t* test). The average BN benefit score for those with hearing aids was 3.44 (SD 17.5; n=9; *P*=.54, 2-tailed dependent *t* test), and the average BN benefit score for those without hearing aids was 20.43 (SD 15.1; n=7; *P*=.01, 2-tailed dependent *t* test). The average benefit score for reverberation was 10.84 (SD 16.95; N=16; *P*=.02, 2-tailed dependent *t* test). The average reverberation benefit score for those without hearing aids was 11.12 points higher than those with hearing aids (*t*_14_=2.14; *P*=.20, 2-tailed independent *t* test). The average reverberation benefit score for those without hearing aids was 17.10 (SD 16.0; n=7; *P*=.03, 2-tailed dependent *t* test), and the average reverberation benefit score for those with hearing aids was 5.98 (SD 17.0; n=9; *P*=.32, 2-tailed dependent *t* test).

Three of our participants requested to continue use of the wristband after the study ended, and hence, they did not fill out the final questionnaire. Of those who did, some had criticisms (“I’m really unsure if the Clarify band was helpful or not”) and some had praise (“It was very beneficial. Thank you”); however, the comments were too few to be statistically meaningful.

## Discussion

In this study, we expanded on our prior work that showed deaf and hard of hearing individuals are capable of identifying sound categories through patterns of vibration applied to the wrist [12]. Here, we demonstrated that individuals with high-frequency hearing loss can improve their subjective understanding of speech communication using vibrational representations of high-frequency speech sounds on the wrist. The results demonstrate that after 1 week of wearing the wristband, participants were able to improve their subjective ability to understand conversations during daily interactions. They then continued to improve, at a slower rate, throughout the 6-week study. This reflects prior research findings of an innate ability for those with hearing loss to rapidly learn to interpret tactile vibrations as a substitute for audio information [25]. The understanding of vibrations is further strengthened and perfected over time with practice as the portions of the auditory cortex that respond to tactile vibration expand [26-28].

We further found that participants who started the study with a higher baseline APHAB score experienced a greater improvement in their subjective ability to understand speech by the end of the 6-week trial. Of 16 participants, 14 ended the study with an APHAB score of 40 or below (which translates to perceived difficulty understanding speech less than half of the time). A total of 5 participants started the study with an unaided APHAB score of 50 points or higher; for 3 of them, the final APHAB benefit score was >30 points. One potential explanation for why participants who started the trial with greater difficulty understanding speech experienced greater improvement is that more of their auditory cortex was available for the interpretation of tactile sound representation [26]. It is also possible that participants who started the study with a higher APHAB score had more room for improvement, as higher APHAB scores indicate a higher degree of perceived disability. This could be an interesting topic for future research.

Participants without hearing aids demonstrated a trend toward higher self-reported benefit from vibrotactile sensory substitution for speech understanding, though this did not reach statistical significance. Given that this group started the study trending toward a higher APHAB score, we presume the difference is because the hearing aid group already benefits from their technology and therefore has less room for improvement. It is difficult to predict what the interaction between hearing aids and vibrotactile feedback will be because of the differing signal processing techniques used in digital hearing aid technologies. Digital hearing aids convert sound waves into numerical codes before amplifying them. This code contains information about a sound’s frequency and amplitude, allowing the hearing aid to be specially programmed to amplify some frequencies more than others. Digital sound processing capabilities allow an audiologist to adjust the hearing aid to a user’s needs and different listening environments. Digital hearing aids can also be programmed to focus on sounds coming from a specific direction. The wristband may represent sounds that differ significantly from those represented by the hearing aid. Future studies can explore directly connecting the wristband to the user’s hearing aids through a Bluetooth signal so that the wristband’s signals directly correspond with the sounds the user is hearing. For this study, the small sample size rendered the study underpowered to detect differences between those who used hearing aids and those who did not at *P*<.05. Future studies will be designed to investigate this finding further.

Individuals with hearing impairment have great difficulty understanding speech in the presence of BN. It is one of the primary complaints expressed by many with hearing loss, and one of the most difficult impairments to resolve. Individuals with hearing loss are unable to resolve the closely spaced harmonics of speech sounds to perform a spectral analysis with enough detail to extract the time-frequency portions of the speech that are relatively spared from corruption by the noise background [29]. In hearing aids, the BN modulators have not been shown to be highly effective at helping in these situations [30]. In this study, we demonstrated that the addition of vibrotactile feedback in the presence of BN enabled individuals who did not wear hearing aids to hear speech communication better based on their subjective experience (Figure 7). Interestingly, the final average BN score for the subgroup without hearing aids was 28.95 (SD 16.15; n=7) and the final average BN score for the subgroup with hearing aids was 40.04 (SD 18.78; n=9), suggesting that those who use hearing aids may benefit from using vibrotactile feedback during conversations with BN instead of using their hearing aids. While our data does not offer conclusive evidence of this due to several limitations, it does offer an area worth further exploration in larger studies.

Reverberation is the persistence of a sound after it is produced and is created when the sound is reflected off of surfaces or objects. It is most noticeable when the source of the sound has stopped, but the reflections continue. As the sound reflects off of surfaces and is absorbed by others, the quality of the sound degrades. Every room or outdoor environment has a different level of reverberation due to the construct of the room or area, the reflectiveness of the materials, and the objects in it. Reverberation is natural to every area, but in areas where the reverberation is very high, it can reduce speech intelligibility, especially when BN is also present. Individuals with hearing loss, including users of hearing aids, frequently report difficulty in understanding speech in reverberant, noisy situations [31]. Most hearing aids, both digital and analog, have limited ability to help individuals with hearing loss in areas of high reverberation [32]. We found that the addition of vibrotactile haptic vibration to the wrist in reverberant environments tended to help the participants without hearing aids more than those with hearing aids, though the difference did not reach statistical significance (Figure 6). One possibility to be tested is that individuals who use hearing aids may find haptic vibrations to be more helpful in reverberant environments when the hearing aids are removed because it would eliminate any conflict between the digital processing of the hearing aid and the vibrational signals that are providing information about the sounds of speech without processing.

In the context of the APHAB, EOC describes the effort involved in communication under relatively easy listening environments. The interesting discovery from our results was that individuals who use hearing aids experienced a significant subjective improvement in their understanding of conversations under easy listening conditions. In easy listening environments where hearing aids help the most and perform the least amount of digital signal processing, the addition of haptic vibrations added the greatest amount of additional benefit. Upon completion of the trial, the average EOC score for the subset of participants who were users of hearing aids was 14.65 (SD 6.99; n=9), indicating little to no subjective difficulty understanding speech in easy listening environments. For the subset of participants who were not users of hearing aids, the average EOC score upon completion of the trial was 16.88 (7.73; n=7). Even without the additional help of hearing aids, these participants ended the study with an equivalent subjective capability for understanding speech in easier listening environments, despite starting the trial with a higher level of disability (Figure 8).

There are limitations to this study. First, the small sample size prevents extrapolation of the results to larger populations; this will be addressed in future studies. We were also limited in our ability to collect speech comprehension data in a noise-controlled environment with standardized volume controls—this is because the testing was done in participant homes instead of a laboratory. As a result, this study depended on self-report data (APHAB), which always has the potential to be influenced by a placebo effect. Another limitation is that some participant audiograms were assessed via phone apps rather than an audiologist’s office; however, it should be noted that these appear to yield roughly equivalent results [5]. We also note that the specific type of hearing loss was not controlled beyond meeting the audiogram requirements. One final thing to note is that participants could move their hand (and, hence, their wristband), meaning that the microphone placement was not standardized in a single position. We do not consider this a limitation of the study, as the study is meant to test whether a vibrotactile wristband can be used to detect sound. The positive results reported here suggest that the mobility of the microphone does not present a problem.

We have demonstrated that vibrotactile sensory substitution helps individuals with high-frequency hearing loss improve their subjective understanding of verbal communication. The device demonstrated here is a wristband that delivers spatially distinguishable vibrations to the wrist in correspondence with high-frequency phonemes. We found that while both hearing aid and non–hearing aid users with high-frequency hearing loss reported a benefit, vibrotactile feedback tended to be more beneficial for non–hearing aid users. However, the small sample size rendered the study underpowered to detect this difference at *P*<.05, and further study is necessary to validate this finding. Finally, our results also demonstrated that those who started the study with a higher APHAB score (greater hearing disability) experienced the greatest amount of benefit from vibrotactile feedback.

## Supplementary material

10.2196/49969 Multimedia Appendix 1 Microphone characteristics.

10.2196/49969 Multimedia Appendix 2 Abbreviated Profile of Hearing Aids Benefit summary statistics per week for all participants, the subgroup that did not wear hearing aids, and the subgroup that did wear hearing aids. The benefit score is the baseline score minus the final score.

10.2196/49969 Multimedia Appendix 3 Abbreviated Profile of Hearing Aids Benefit subscale summary statistics per week for all participants, the subgroup that did not wear hearing aids, and the subgroup that did wear hearing aids.

## Abbreviations

APHAB: Abbreviated Profile of Hearing Aid Benefit
BN: background noise
EOC: ease of communication
GRMS: root mean squared acceleration from gravity
LRA: linear resonant actuator
