# Supplementary material for: Cross-Modal Sensory Boosting to Improve High-Frequency Hearing Loss: Device Development and Validation
Source: JMIRx Med. 2024 Feb 9;5:e49969. doi: 10.2196/49969 (PMC11008433; doi:10.2196/49969)
Supplement: Multimedia Appendix 1 [file xmed-v5-e49969-s001.pdf]

Frequency range (Hz)	300 to 7500
Reference test frequency (kHz)	1
Harmonic distortion reference (SPL)	105
Harmonic distortion (%)	.2
Directionality	Omni
Equivalent input noise (dBA SPL)	29
Dynamic range (dB)	91
Acoustic overload point (dB SPL)	120
Absolute max (dB SPL)	160
Sensitivity levels[3]	3
Feedback suppression	Yes
Ambient noise suppression	Yes
Frequency range adjustable	Yes

Table S1. Microphone characteristics
