# Supplementary material for: Cross-Modal Sensory Boosting to Improve High-Frequency Hearing Loss: Device Development and Validation
Source: JMIRx Med. 2024 Feb 9;5:e49969. doi: 10.2196/49969 (PMC11008433; doi:10.2196/49969)
Supplement: Multimedia Appendix 2 [file xmed-v5-e49969-s002.pdf]

	Baseline	Week 1	Week 2	Week 3	Week 4	Week 5	Week 6	6 Week Change
Average	40.32	31.71	29.60	31.37	29.58	27.96	27.93	-11.95
Standard Deviation	15.38	10.35	13.21	14.06	12.67	11.56	12.00	6.20
Min	20.06	13.89	9.83	8.56	7.28	5.39	5.44	-24.28
Max	70.78	51.44	53.50	59.67	52.06	52.06	52.06	-5.50
Range	50.72	37.56	43.67	51.11	44.78	46.67	46.61	18.78
Standard deviation with HA	14.61	8.97	12.54	11.50	10.89	10.40	12.02	6.11
Min with HA	22.83	21.50	15.33	14.50	13.78	17.94	13.78	-24.28
Max with HA	57.50	47.17	52.78	50.61	51.39	52.06	52.06	-5.61
Range with HA	34.67	25.67	37.44	36.11	37.61	34.12	38.28	18.67
Standard deviation without HA	16.66	12.67	15.01	17.82	15.60	13.78	12.51	6.60
Min without HA	20.06	13.89	9.83	8.56	7.28	5.39	5.44	-22.94
Max without HA	70.78	51.44	53.50	59.67	52.06	42.94	40.28	-5.50
Range	50.72	37.56	43.67	51.11	44.78	37.56	34.83	17.44

Table S2. APHAB summary statistics per week for all participants, the subgroup that did not wear hearing aids and the subgroup that did wear hearing aids. The benefit score is the baseline score minus the final score.
