# Supplementary material for: Cross-Modal Sensory Boosting to Improve High-Frequency Hearing Loss: Device Development and Validation
Source: JMIRx Med. 2024 Feb 9;5:e49969. doi: 10.2196/49969 (PMC11008433; doi:10.2196/49969)
Supplement: Multimedia Appendix 3 [file xmed-v5-e49969-s003.pdf]

	Baseline			Week 6			Week 6 Benefit Score		
	EOC	BN	RV	EOC	BN	RV	EOC	BN	RV
Average	31.06	46.06	43.84	15.63	35.19	33.00	15.44	10.88	10.84
Standard Deviation	17.04	17.25	18.31	7.16	18.02	14.92	13.88	17.54	16.95
Min	1.00	14.50	16.67	1.00	6.83	6.67	0.00	-35.50	-16.67
Max	64.50	79.17	77.00	27.00	68.83	60.33	48.33	48.00	49.83
Range	34.71	49.38	48.17	16.88	28.95	31.07	17.83	20.43	17.10
Average without HA	28.22	43.48	40.48	14.65	40.04	34.50	13.57	3.44	5.98
Standard deviation without HA	19.17	16.12	16.39	6.99	18.78	14.83	11.85	15.12	15.95
Min without HA	20.67	18.67	16.67	2.83	6.83	6.67	4.33	6.00	-2.17
Max without HA	64.50	79.17	77.00	25.00	52.17	50.00	41.67	48.00	49.83
Range without HA	43.83	60.50	60.33	22.17	45.33	43.33	37.33	42.00	52.00
Average with HA	14.45	19.35	21.01	7.73	16.15	15.98	13.88	17.54	16.95
Standard deviation with HA	19.17	16.12	16.39	6.99	18.78	14.83	15.72	16.22	16.95
Min with HA	1.00	14.50	25.00	1.00	12.50	12.50	0.00	-35.50	-16.67
Max with HA	62.50	70.67	68.50	27.00	68.83	60.33	48.33	24.83	43.50
Range with HA	61.50	56.17	43.50	26.00	56.33	47.83	48.33	60.33	60.17

Table S3. APHAB subscale summary statistics per week for all participants, the subgroup that did not wear hearing aids and the subgroup that did wear hearing aids. EOC = Ease of Communication. BN = Background Noise. RV = Reverberation
